# EFFECTS OF AN INTERGENERATIONAL EXERCISE BUDDY PROGRAM ON PARTICIPANTS

**DOI:** 10.1093/geroni/igz038.3197

**Published:** 2019-11-08

**Authors:** Eun hae Kim, Kyong Hee Chee, Clay DeStefano, Anna Broome

**Affiliations:** 1 Texas State University, San Marcos, Texas, United States; 2 Greater San Marcos Area Seniors Association/Price Center, San Marcos, Texas, United States; 3 SMTX Yoga, San Marcos, Texas, United States

## Abstract

Structured social support may enhance the benefits of an exercise class for participants. This study examined the effects of an intergenerational exercise buddy program on participant well-being. A convenience sampling was used to recruit participants from Central Texas (N = 51): 34 were community-dwelling adults aged 65 and above, and 17 were university students aged 18-25 years. The study used a pretest-posttest, quasi-experimental design and focus group interviews. Randomly selected 18 older-adult participants were paired with young-adult participants as an exercise buddy for each other to attend 8 weekly Tai Chi or Chair Yoga classes at a community center. Meanwhile, 16 older-adult participants formed a control group attending different exercise classes without young-adult buddies. The survey results show that, compared to the control group, the intervention group (n = 35) had significantly greater satisfaction with life (p < .09 ) and a more positive attitude towards aging (p < .01) after attending exercise classes with their buddies. In focus group interviews, the participants most frequently mentioned that commitment to their buddies as a key factor for class attendance. The participants typically perceived that their buddies were pleasant and did not judge or treat them based on their age. They stressed the positive effects of building relationships with all involved in the program, including the exercise instructors. Meanwhile, control-group participants wished that they, too, had buddies. Although the study should be replicated with a larger sample, its findings suggest that an intergenerational exercise program offers added benefits for participants.

